# Toward the precision breast cancer survival prediction utilizing combined whole genome-wide expression and somatic mutation analysis

**DOI:** 10.1186/s12920-018-0419-x

**Published:** 2018-11-20

**Authors:** Yifan Zhang, William Yang, Dan Li, Jack Y Yang, Renchu Guan, Mary Qu Yang

**Affiliations:** 1 MidSouth Bioinformatics Center and Joint Bioinformatics Ph.D. Program of University of Arkansas at Little Rock and Univ. of Arkansas Medical Sciences, 2801 S. Univ. Ave, Little Rock, 72204 USA; 2 Department of Computer Science, Carnegie Mellon University School of Computer Science, 5000 Forbes Ave, Pittsburgh, 24105 USA

**Keywords:** Breast Cancer, Somatic mutations, Whole genome-wide expression, Survival analysis, Precision survival prediction

## Abstract

**Background:**

Breast cancer is the most common type of invasive cancer in woman. It accounts for approximately 18% of all cancer deaths worldwide. It is well known that somatic mutation plays an essential role in cancer development. Hence, we propose that a prognostic prediction model that integrates somatic mutations with gene expression can improve survival prediction for cancer patients and also be able to reveal the genetic mutations associated with survival.

**Method:**

Differential expression analysis was used to identify breast cancer related genes. Genetic algorithm (GA) and univariate Cox regression analysis were applied to filter out survival related genes. DAVID was used for enrichment analysis on somatic mutated gene set. The performance of survival predictors were assessed by Cox regression model and concordance index(C-index).

**Results:**

We investigated the genome-wide gene expression profile and somatic mutations of 1091 breast invasive carcinoma cases from The Cancer Genome Atlas (TCGA). We identified 118 genes with high hazard ratios as breast cancer survival risk gene candidates (log rank *p* <  0.0001 and c-index = 0.636). Multiple breast cancer survival related genes were found in this gene set, including *FOXR2*, *FOXD1*, *MTNR1B* and *SDC1*. Further genetic algorithm (GA) revealed an optimal gene set consisted of 88 genes with higher c-index (log rank *p* <  0.0001 and c-index = 0.656). We validated this gene set on an independent breast cancer data set and achieved a similar performance (log rank *p* <  0.0001 and c-index = 0.614). Moreover, we revealed 25 functional annotations, 15 gene ontology terms and 14 pathways that were significantly enriched in the genes that showed distinct mutation patterns in the different survival risk groups. These functional gene sets were used as new features for the survival prediction model. In particular, our results suggested that the Fanconi anemia pathway had an important role in breast cancer prognosis.

**Conclusions:**

Our study indicated that the expression levels of the gene signatures remain the effective indicators for breast cancer survival prediction. Combining the gene expression information with other types of features derived from somatic mutations can further improve the performance of survival prediction. The pathways that were associated with survival risk suggested by our study can be further investigated for improving cancer patient survival.

**Electronic supplementary material:**

The online version of this article (10.1186/s12920-018-0419-x) contains supplementary material, which is available to authorized users.

## Background

Breast cancer is the most commonly occurring female cancer in developed countries. Over 40,000 breast cancer deaths and approximately 250,000 new cases were reported in 2016 [1]. The survival rate in HER2+ breast cancer patients [2] has been remarkably increased through targeted therapies including tyrosine kinase inhibitors. Adjuvant treatments such as chemotherapy also improved the 5-year survival rate of the breast patients [3, 4]. However, the significant side effects of chemotherapy can shorten the lifespan of cancer patients in some cases [5]. Additionally, due to potential metastasis and invasion of cancer, the overall outcome for breast cancer patients remains bleak. An effective survival predictor, which is capable of helping cancer treatment and foreseeing the clinical outcomes, can improve life quality and lifespan of cancer patients. Thus, better prognostic biomarkers of survival risk prediction are needed.

In clinical practice, clinicopathological prognostic indicators, such as tumor size, lymph node (LN) status and pathological grade [6, 7] have been widely used in prognostic analysis models. However, in some cases, the treatment responses vary greatly even with similar prognoses. Recently, single cell genomics analysis have shown that ostensibly similar tumor defined by traditional pathological analysis could be distinct diseases at the cell levels [8–10]. Due to the highly heterogeneous nature of cancer cells, the predictive ability of some traditional indicators can be less effective for over 50% of patients [11].

Multiple survival analysis models have developed mainly based on gene expression profiles. The cox regression model and machine learning algorithms have been widely used to reveal molecular signatures related to survival. Bair et al. [12] used semi-supervised methods to cluster patients with different survival risk based on gene expression profiles and clinical data. Sun et al. [13] applied univariate Cox regression analysis and identified nine long noncoding RNAs (lncRNAs) that were highly associated with their metastasis-free survival for breast cancer patients. Zhang et al. [14] developed a two-stage method, using Bayesian hierarchical Cox model and the penalized Cox model, and incorporated pathway information with gene expression profiles to predict survival.

Most survival prediction methods mainly utilize gene expression profiles. Somatic mutations are involved in the cancer development [15]. Several studies showed that genetic mutations are associated with cancer survival, such as BRCA1- and BRCA2-related mutations, and HER2 somatic mutations [16, 17]. Thus, identifying survival related mutations are meaningful for prognosis and treatment. However, it is difficult to detect mutation patterns in a patient cohort for survival analysis since most commonly mutated genes are found in less than 10% of patients. Consequently, using common mutations alone to predict survival is less effective. To address this issue, we integrated somatic mutations with pathway, function annotation and gene ontology (GO) analysis. We then synergistically used the significantly mutated gene sets as predictors coupled with gene expression for survival analysis. Our results suggested that combining somatic mutations improved the performance of survival risk prediction.

## Results

### Gene differential expression and survival risk detection

**Fig. 1:**
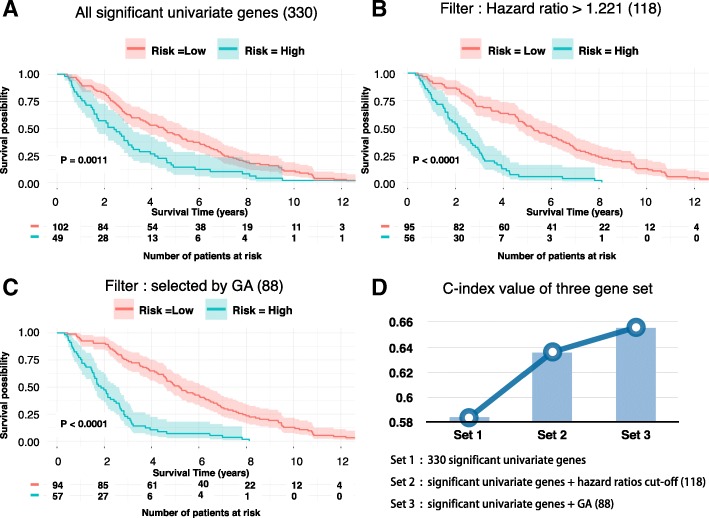
The survival analysis using different type of features. The Kaplan–Meier curves for 330 significant univariate genes (**a**), 118 significant univariate genes with hazard ratios higher than 1.221(**b**), 88 genes that select by GA (**c**). **d** showed the C-index for these three models

We identified 4327 differentially expressed genes (adjust P-value < 0.01 and |logFC| > 2) based on the gene expression profile for 1091 breast cancer patients (151 deceased and 940 living) and 113 normal tissue samples, using the software tools developed in our laboratory (http://mqyang.net) along with other existing tools. Next, we applied univariate cox regression analysis to select the differential expressed genes that were also associated with survival. Out of 4327 genes, 330 were significantly associated with survival time (P-value <  0.05). The patients were clustered into two distinct groups, high survival risk and low survival risk, using k-means according to the expression levels of 330 survival-related genes. Then, a Cox proportional hazards model was fitted to the patient survival data for the two groups (Fig. 1a). We calculated the Concordance index (C-index) to assess the predictive ability of our survival model. The C-index measures the concordance between the observed survival times and predicted survival times. The C-index of 330 gene signature-based model was 0.584 (P-value = 0.0011).

Out of the 330 genes, we further selected 118 genes with hazard ratio > 1.221. The resulting new prediction model yielded a higher C-index, 0.636.(P-value < 0.001). According to Kaplan–Meier curve (Fig. 1b), the survival rate of 1500 days (4 years and 1 month) was 63.2% and 12.5% for patients in the low risk group and high risk group, respectively (Fig. 1b). The 118 genes contained several well-known breast cancer survival related genes or oncogenes, including *FOXR2, FOXD1, MTNR1B* and *SDC1* [18–24]. Additionally, PANTHER [25] pathway analysis revealed 17 biological processes that were significantly enriched in the 118 genes (Additional file 1). Some are known biological process related to cancer, such as: DNA replication (P-value = 0.037), structural molecule activity (P-value = 0.00035), system development (P-value =0.00047), and cytoskeleton (P-value 0.000062) [26–28], while some others have not  been well studied (Additional file 1), such as peptide cross-linking (P-value=0.00027).

### Survival-related genes selected by genetic algorithm

**Fig. 2:**
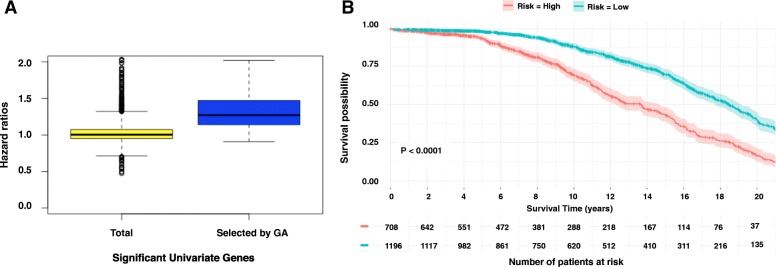
The hazard ratio values and the Kaplan–Meier estimator for an independent patient dataset. **a** The hazard ratios of 330 significant univariate genes (yellow) and 88 genes (blue) selected by GA. **b** The Kaplan–Meier curve using 61 of 88 GA selected genes for a METABRIC breast cancer patient dataset

We developed a genetic algorithm (GA)-based method to further optimize gene selection from original 330 significant univariate genes. The method is able to assess the combinatorial effects of multiple genes on survival. As a result, 88 genes were revealed as the optimal gene set which maximized the C-index. Using this gene set, we conducted survival analysis (Fig. 1c) and achieved a better c-index 0.656 compared to 0.636 yielded by the model constructed based on the 118 genes selected by hazard ratio. We found that 67 of 88 (75.3%) genes were overlapped with the 118-gene set. Hence, hazard ratio from the univariate test may not be the only factor that determines the prediction accuracy. Nevertheless, genes with higher hazard ratio tend to have higher possibility to generate more accurate survival prediction. (Fig. 2a).

We obtained an independent data set from Molecular Taxonomy of Breast Cancer International Consortium (METABRIC) [29] to validate our models. This dataset includes the expression profile and clinical data for 2509 breast cancer tissue samples. We found 61 genes in the 88-gene set and 64 genes in the 118-gene set shown in the expression profile of this patient cohort. Similar data normalization and survival analysis were performed. The models based on both gene sets were able to separate the high-risk and low-risk survival groups (*P*-value < 0.0001, Fig. 2b and Additional file 2). The C-index was 0.614 and 0.6004 for the models based on 61 GA-selected genes and 64 hazard-ratio-selected genes, respectively. Consistent with the result for the TCGA dataset, the gene set that was identified by GA searching yielded higher prediction accuracy.

### Survival-related somatic mutations

**Fig. 3:**
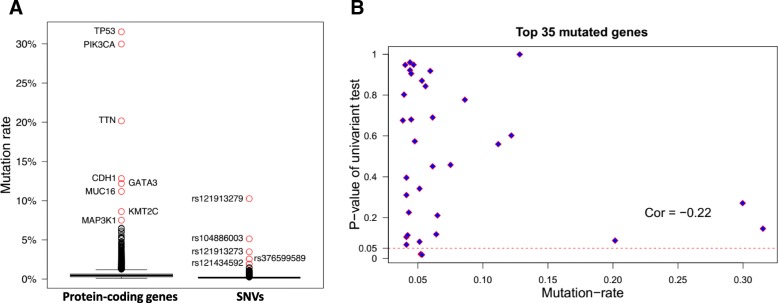
Distribution of mutation rates and the correlation with univariate P-values. **a** The distribution of mutation rates for genes and SNPs in the TCGA breast cancer patients. **b** The correlation between univariate *P*-value and mutation rate for top 35 mutated genes

A total of 105,425 single-nucleotide variances (SNVs) were identified in 1044 breast cancer patient samples (1044 of 1091 patients have mutation data). The maximum and median SNV mutation rate was 10.4% and 0.095%, respectively (Fig. 3a), while the maximum and median gene mutation rate was 32.95% and 0.48%, respectively. rs121913279 was the most frequent SNP, which was located at the *PIK3CA* gene. *TP53*, *PIK3CA*, *TTN*, *CDH1*, *GATA3*, *MUC16*, *KMT2C* and *MAP3K1* were the top frequently mutated genes (Fig. 3a). The gene pairs consisting of these eight genes have differential mutation patterns, four pairs tend to mutual exclusively, while six pairs tend to be co-occurred (P-adjust <= 0.008) (Table 1). The C-index and *P*-value of the survival model based on these eight top mutated genes were 0.539 and 0.085. We expanded the mutated gene list to 88, 118, 330, which matched the number of differential genes used in the survival models. The resulting c-index for these different gene signature set was 0.565, 0.522 and 0.567, and corresponding P-value was 0.035, 0.23 and 0.028. Thus, the top mutated genes were not necessarily effective gene signatures for survival prediction; the correlation coefficient of gene mutation rates and P-values of the univariate tests was − 0.22 (Fig. 3b).

**Table 1:** The top mutated genes in the TCGA 1044 breast cancer dataset

Gene A	Gene B	A Not B	B Not A	Both	Log Odds Ratio	Adjusted *p*-Value	Tendency
*TP53*	*CDH1*	338	125	9	−2.067	< 0.001	Mutual exclusivity
*TP53*	*GATA3*	336	116	11	−1.771	< 0.001	Mutual exclusivity
*TP53*	*MAP3K1*	336	79	11	−1.327	< 0.001	Mutual exclusivity
*TP53*	*PIK3CA*	265	266	82	−0.641	< 0.001	Mutual exclusivity
*PIK3CA*	*CDH1*	284	70	64	0.735	0.002	Co-occurrence
*TP53*	*TTN*	264	104	83	0.62	0.004	Co-occurrence
*PIK3CA*	*KMT2C*	296	55	52	0.75	0.006	Co-occurrence
*TTN*	*KMT2C*	154	74	33	0.846	0.007	Co-occurrence
*PIK3CA*	*MAP3K1*	303	45	45	0.798	0.008	Co-occurrence
*TTN*	*MUC16*	146	68	41	1.209	< 0.001	Co-occurrence

To combine gene expression and mutation for survival analysis, we first clustered the breast cancer patients into two groups (high survival risk and low survival risk) based on the expression levels of 118 genes. Then we analyzed the corresponding somatic mutation profiles for the patients in the distinct groups. We found that 48,404 and 57,024 SNVs were found for the patients in the high-risk group and the low risk group, respectively.

**Fig. 4:**
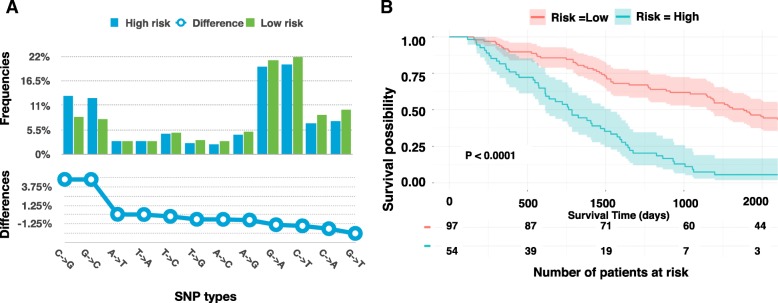
The mutation of different SNP types. **a** The frequencies of different SNP types in the high and low risk groups. **b** The Kaplan–Meier curve for using C → G and G → C SNP frequency combine with 118 gene expression as indicators

We compared the percentage of different types of SNVs in the two groups (Fig. 4a). The C- > G and G- > C SNVs frequencies in the high-risk group were significantly higher than these SNVs (13.1% versus 8.31%, 12.6% versus 7.82%) in the low risk group (t-test, P-value = 5.5e-14, P-value = 1.3e-14). We thus used C- > G and G- > C SNP frequencies as the two features to perform survival analysis. The survival risk curves showed a noticeable yet insignificant difference ( P-value= 0.36). Combining the 118 gene expression with C- > G and G- > C SNP frequencies, we conduct another survival analysis. We obtained similar prediction accuracy as using the gene expression alone (Fig. 4b). Our results suggested that GC SNV frequencies were weaker features for survival prediction.

We also calculated the mutation rates of all genes in the high-risk and low-risk groups. The mutation rate here refers to the percentage of the patients who harbored somatic mutation(s) in a specific gene in the patient group. Then we computed the mutation rate differential scores (MRDS)(Method) for each individual genes.

Some genes showed quite distinct mutation rates in the different groups. For example, the mutation rates of *TP53* in the high and low risk group were 0.634 and 0.208, respectively. We selected the top 2000 genes ranked by MRDS. Multiples genes in this list present in breast cancer related pathways. For example, *ATM, ATR, TP53, PTEN, CASP8, and IGFBP3* participate in p53 signal pathway, while *MMP, EGFR, CREP, PLC, and RAS* participate in estrogen signaling pathway.

Based on DAVID analysis [30, 31], we identified 30 pathways, 114 functional annotations and 38 Gene Ontology (GO) terms that were significantly enriched of the 2000 mutated genes (*P*-value < 0.05). We then build new features using these molecular function gene sets. If a patient carried mutation(s) in at least one gene of individual enriched function gene sets, we set the corresponding feature value as 1; otherwise we set the value as 0. Then we performed a univariate Cox regression analysis for each of new features. As a result, we identified 15 function annotations (FA), 15 GO terms and 14 pathways that were significant in the univariate test (*P*-value < 0.05 and hazard ratio > 1.221). Using a total of 54 mutated functional gene set, the C-index survival model was 0.591 (P-value = 0.0016). The C-index of the models that utilized different type of gene set (Table 2) showed that integrating gene expression and mutation analysis generated a survival model with better accuracy (Table 2) (Fig. 4b).

**Table 2:** C-index of the Cox proportional hazards models based on different features

Feature type	Features	C-index	*P*-value
Gene expression	330 significant univariate genes	0.584	0.0011
	118 significant univariate genes with hazard ratio > 1.221	0.636	< 0.0001
	88 significant univariate genes selected by GA	0.656	< 0.0001
Somatic mutation	25 functional annotations	0.603	0.0012
	15 gene ontology terms	0.567	0.0013
	14 pathways	0.548	0.0037
	54 functional gene sets combining functional annotations, GO terms and pathways	0.591	0.0016
Gene expression & somatic mutation	All 142 features (88 significant univariate genes and 54 functional gene sets)	0.658	< 0.0001

## Discussion

The gene signatures derived from expression profiles have commonly been used in survival analysis [18–21, 24]. The survival-related genes are often selected through the univariate regression model. The resulting P-value and/or hazard ratio were used as criteria for gene selection. However, the gene list composed of the gene selected from the univariate tests may not be necessary the optimal gene set for the survival prediction. To address this limitation, we employed a GA searching algorithm to optimize gene set selection for accurate survival prediction. The GA searching enables the assessment of the combinatorial effect of a gene set on the survival analysis. As expected, we identified a set of genes that yielded higher prediction accuracy than the gene list obtained from the univariate regression model. In this study, the GA only searched for the best gene combination of 330 univariate genes, which was the length of initial chromosome. The pre-filtered gene candidate set can reduce the searching space of the GA algorithm and save the computation time, however, the optimal gene set could be overlooked by this strategy.

**Fig. 5:**
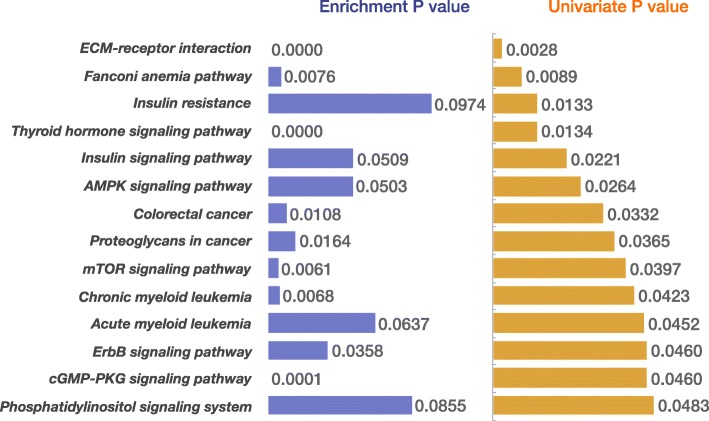
The 14 pathways enriched by differential mutated genes. The 14 pathways were significantly enriched by the top 2000 differentially mutated genes with higher MRDS (blue bar). These pathways also showed significance in the Cox univariate regression test (yellow bar)

The cancer survival analysis merely using the gene expression levels shows the limited ability for accurate survival risk prediction [32]. The genetic mutations have an important role in cancer development. Hence, the genetic mutation profiles can provide additional values for survival analysis and lead to uncover genetic variations that are associated with patient survival [27]. We found that the most frequently mutated genes were not necessarily significant in survival analysis. The correlation coefficient of the top eight mutated genes and the *P*-values from the univariate regression model is − 0.22 in the breast cancer dataset that we studied. The mutated genes selected by the univariate regression test often resulted in very unbalanced survival risked groups (Additional file 3). In addition, it is commonly known that the drive gene mutations in a pathway tend to be mutually exclusive. Here, we proposed a system biology method: first projected the somatic mutations onto the pathways and gene function sets, then used significantly mutated pathways or function gene sets as additional signatures for survival analysis. For example, we found 14 pathways were significant abundance of the mutated genes as well as showed significance in the univariate regression test (Fig. 5). The mutated rates of these pathway-genes were relatively low (Additional file 4).

Despite of low mutation rate of pathway-gene, they tended to be more effective for survival prediction. In the Cox univariate regression, the 14 pathways consistently demonstrated lower *P*-values than the top mutated genes set (Additional file 5). Here, the number of top mutated genes set is the same as the number of mutated genes in the corresponding pathways. We also performed multivariate regression test. In the multivariate test, each individual mutated gene in the pathways or the top mutated genes was considered a feature. We found most pathway-genes tend to have high P-value than the individual top mutated genes, excepting the Fanconi anemia pathway and ECM-receptor interaction pathways. Specially, the Fanconi anemia pathway performed well in both univariate regression and multivariate regression models (Additional file 5), suggest this pathway has an essential role in the breast cancer survival.

In this study, we integrated somatic mutations with gene expression in our survival analysis and built an improved model for survival risk prediction. Further improvement could be achieved when several related issues are solved. First, previous studies showed that somatic mutation identification remains inaccurate [33, 34]. The mutations identified by different mutation callers often have relatively low overlap. Better mutation caller can potentially improve survival prediction accuracy. Second, somatic mutations are highly heterogeneous among patients. Identifying survival driver genes could be valuable for survival prediction. In addition, allele frequency, the mutation positions and the other mutation information can be further incorporated into the survival model for survival model enhancement. Unlike gene expression, the quantitative relationship between somatic mutation and phenotypic characteristics are unknown. With better understanding of somatic mutation roles in the patient survival, we can build a better prognosis model for survival analysis.

## Conclusions

Our study suggested that expressions of genes were the effective indicators for breast cancer survival prediction. By coupling with somatic mutations, we were able to improve the survival predict model, however, the improvement was marginal. Also, we found that some genes demonstrated significantly distinct mutation rates between high risk and low risk breast cancer patients. Better utilizing the mutation difference for cancer prognostic analysis can be further investigated.

## Methods

### Breast cancer datasets

We obtained RNAseq datasets including raw counts and FPKM (Fragments Per Kilobase of transcript per Million) counts from TCGA (The Cancer Genome Atlas) project. The RNAseq datasets were generated from 1091 breast tumor tissue samples. We downloaded the somatic mutation profile generated by MuTect2 workflow, for 1044 tumor tissue samples. Out of 1044 cases in the somatic mutation file, 980 were common cases with gene expression profiles. Survival information was obtained from the metadata for each individual cases. All TCGA datasets used in the study are publicly available at the GDC Portal website (https://portal.gdc.cancer.gov/).

An independent breast cancer datasets including the patient clinical information and the expression profile for 2509 breast cancer patients were downloaded from *cBioportal* (www.cbioportal.org/). The data was generated by the Molecular Taxonomy of Breast Cancer International Consortium (METABRIC).

An R-package ‘edgeR’ was applied for differential expression analysis. We used adjusted P-value lower than 0.01 and absolute log fold change larger than 2 to define significantly differentially expressed genes.

### Feature selection

We first used the univariate Cox regression analysis to choose features for survival analysis. The output of the regression analysis includes three important statistics: statistical significance (p), regression coefficients (coef) (Eq. 1), and hazard ratios (HR) (Eq. 2). These statistics were used to select the significant survival-related features.

1 $$ coef=\frac{n\left(\sum xy\right)-\sum x\sum y}{\sqrt{\left[n\sum {x}^2-{\left(\sum x\right)}^2\right]\left[n\sum {y}^2-{\left(\sum y\right)}^2\right]}} $$

2 $$ HR=\exp \left(\mathrm{coef}\right) $$

where x and y are two vectors that represent the survival time and one predictor variable. Base on C-index statistics, we chose the thresholds P-value  <  0.05 and hazard ratios > 1.221 for selecting survival-related genes.

### Survival analysis

We normalized each feature gene-wisely using the following equation:

3 $$ \frac{g_{i,j}-\underset{j\in N}{\min }{g}_{i,j}}{\underset{j\in N}{\max }{g}_{i,j}-\underset{j\in N}{\min }{g}_{i,j}}\kern2em i=1,2,\dots, M $$

where *g*_*i*, *j*_ represent the value of feature i in the sample j. N is the total number patient samples and M is the total number of features. Then, we used k-means algorithm to identify high survival risk and low survival risk groups. Subsequently, each individual samples were labeled accordingly. Using R package “survival”, the Cox proportional hazards model was fitted with the data and the corresponding C-index was calculated. The R package ‘survminer’ was used to plot the Kaplan–Meier curve.

### Genetic algorithm identifies survival indicators

The genetic algorithm (GA) is a heuristic method for searching the global optimum. We employed the GA to reveal a gene set that yields the best survival prediction accuracy. An R package “Genalg” was used for GA searching. [35]. We set the population size as 200. The chromosome is a 0/1 vector with the length of 330. Here, 330 is the total number of significant univariate genes, 1 means the corresponding gene is selected while 0 refer to the unselected gene. Mutate rate was set to 0.01. The fitness score was calculated by (1 - C-index) of each candidate gene set. The number of generation is set to 100.

### Building somatic mutation and functional gene set based features

Based on the gene signatures derived from the expression profiles, we divided the patients into high and low survival risk groups. Then, the somatic mutation profiles for patients in each group were extracted from the annotated somatic mutation file accordingly. For SNPs, we calculated and compared the percentage of various types of SNPs in the two groups. The t-test was applied to assess whether a type of SNPs demonstrated significantly different mutation rate between the two survival risk groups.

For mutated genes, we compared the mutation rate of each gene (i) in the two groups. Mutation rate (R) was calculated using the number of the samples containing the mutated gene divided by the total number of samples in the individual groups. Then, we calculated a mutation rate differential score (MRDS) for each gene (Eq. 4). The top 2000 genes with higher MRDS were selected for enrichment analysis.

4 $$ \kern2.25em {\boldsymbol{MRDS}}_{\boldsymbol{i}}=\frac{{\boldsymbol{R}}_{\left(\boldsymbol{high},\mathrm{i}\right)}}{{\boldsymbol{R}}_{\left(\boldsymbol{low},\mathrm{i}\right)}}\times {\boldsymbol{e}}^{{\boldsymbol{R}}_{\left(\boldsymbol{high},\boldsymbol{i}\right)}-{\boldsymbol{R}}_{\left(\boldsymbol{low},\boldsymbol{i}\right)}} $$

The DAVID [30, 31] analysis was used to identify functional annotation sets, gene ontology terms and pathways that were enriched by the top differentially mutated genes.

## Additional files

Additional file 1: PATHER significantly enriched terms base on 118 survival related genes. (DOCX 15 kb)

Additional file 2: The Kaplan–Meier estimator for an independent patient dataset. The survival curve base on 64 of 118 hazard-ratio-selected genes for METABRIC breast cancer patient dataset. (PDF 119 kb)

Additional file 3: The Kaplan–Meier estimator using top 35 mutated genes. The Kaplan–Meier curves using 35 mutated genes selected by the univariate regression as predictors displayed two very unbalanced survival risk groups. (PDF 15 kb)

Additional file 4: The comparison of mutation rates for top mutated genes and 14 pathway genes. The mutation rates for 15 different gene sets, including one top-35-mutated gene set and 14 pathway-based gene sets. The genes in each set were ordered by their mutation rates. (PDF 36 kb)

Additional file 5: The *P*-value of the Cox univariate regression analysis using different gene sets. (A) The *P*-values of the Cox univariate regression analysis for pathway-based genes (blue) and top mutated genes (orange). (B) The P-values of univariant test and multivariant test for pathway-based genes (blue and grey) and top mutated genes (orange and purple). (PDF 344 kb)

## Abbreviations

C-Index: Concordance Index
GA: Genetic Algorithm
GO: Gene ontology
LN: Lymph node
LOOCV: Leave-one-out cross-validated
METABRIC: Molecular Taxonomy of Breast Cancer International Consortium
SNPs: Single-Nucleotide Polymorphisms
TCGA: The Cancer Genome Atlas
TKIs: Tyrosine Kinase Inhibitors
