# Supplementary material for: Toward the precision breast cancer survival prediction utilizing combined whole genome-wide expression and somatic mutation analysis
Source: BMC Med Genomics. 2018 Nov 20;11(Suppl 5):104. doi: 10.1186/s12920-018-0419-x (PMC6245494; doi:10.1186/s12920-018-0419-x)
Supplement: Supplementary file 1 — PATHER significantly enriched terms base on 118 survival related genes. (DOCX 15 kb) [file 12920_2018_419_MOESM1_ESM.docx]

**PATHER significantly enriched terms base on 118 survival related gene sets**

| Type | Terms | P-Value |
| --- | --- | --- |
| GO-Slim Molecular function | **structural molecule activity** | 3.50E-04 |
| Pathways | **DNA replication** | 3.70E-02 |
| GO-Slim Biological Process | cell differentiation | 9.90E-06 |
| GO-Slim Biological Process | **system development** | 4.70E-04 |
| GO-Slim Biological Process | cellular protein modification process | 1.30E-04 |
| GO Biological process | peptide cross-linking | 2.70E-04 |
| GO Biological process | Keratinization | 3.40E-04 |
| GO Biological process | keratinization -> keratinocyte differentiation | 3.90E-04 |
| GO Biological process | epidermal cell differentiation -> epithelial cell differentiation | 6.00E-04 |
| GO Biological process | keratinocyte differentiation -> skin development | 3.47E-05 |
| GO Cellular Component | **cytoskeleton** | 6.20E-05 |
| GO Cellular Component | cornified envelope | 2.63E-11 |
