# Supplementary figures and images for: Toward the precision breast cancer survival prediction utilizing combined whole genome-wide expression and somatic mutation analysis
Source: BMC Med Genomics. 2018 Nov 20;11(Suppl 5):104. doi: 10.1186/s12920-018-0419-x (PMC6245494; doi:10.1186/s12920-018-0419-x)

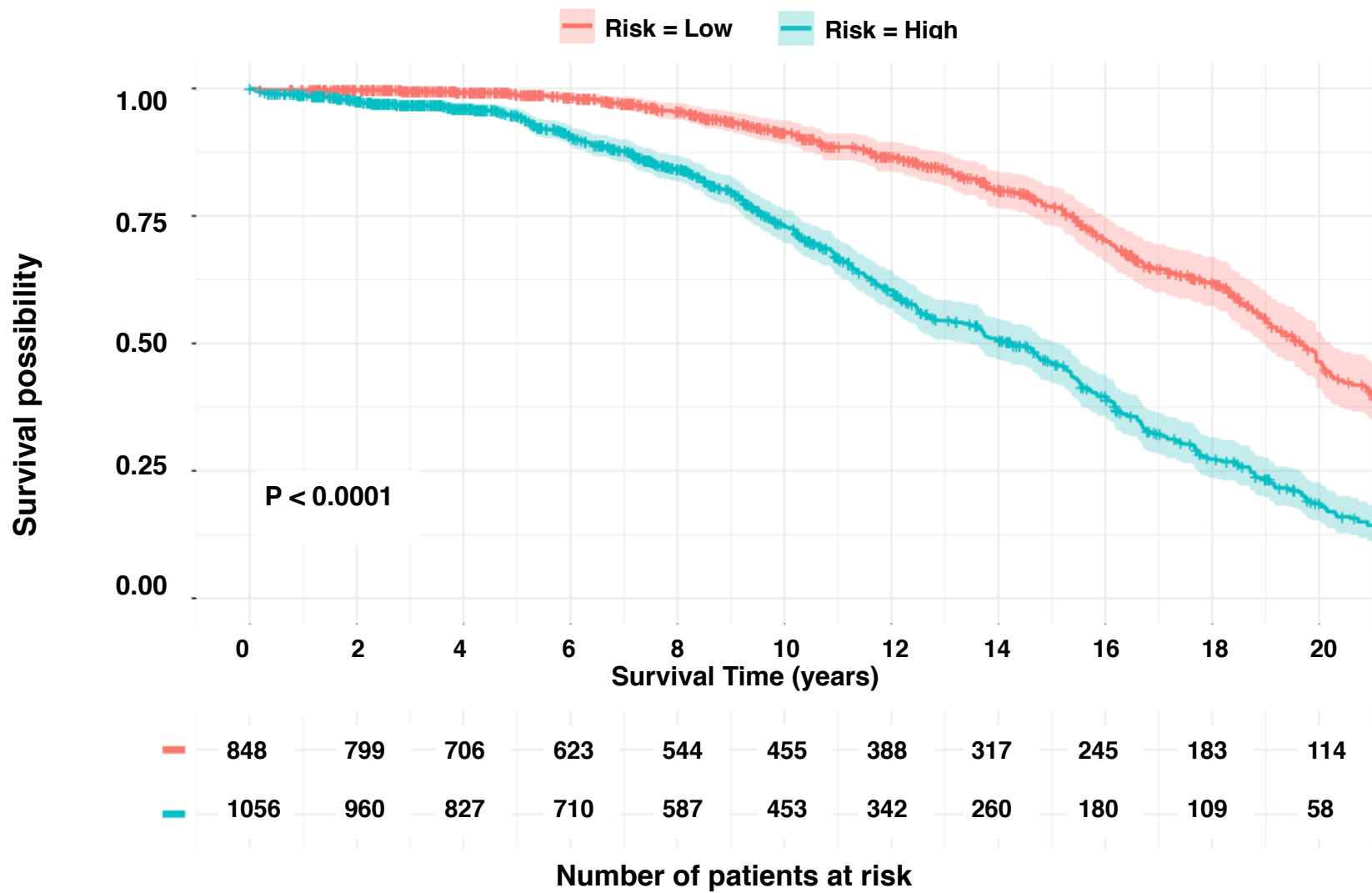

Supplement: Supplementary file 2 — The Kaplan–Meier estimator for an independent patient dataset. The survival curve base on 64 of 118 hazard-ratio-selected genes for METABRIC breast cancer patient dataset. (PDF 119 kb) [file 12920_2018_419_MOESM2_ESM.pdf]

Strata risk=1 risk=2

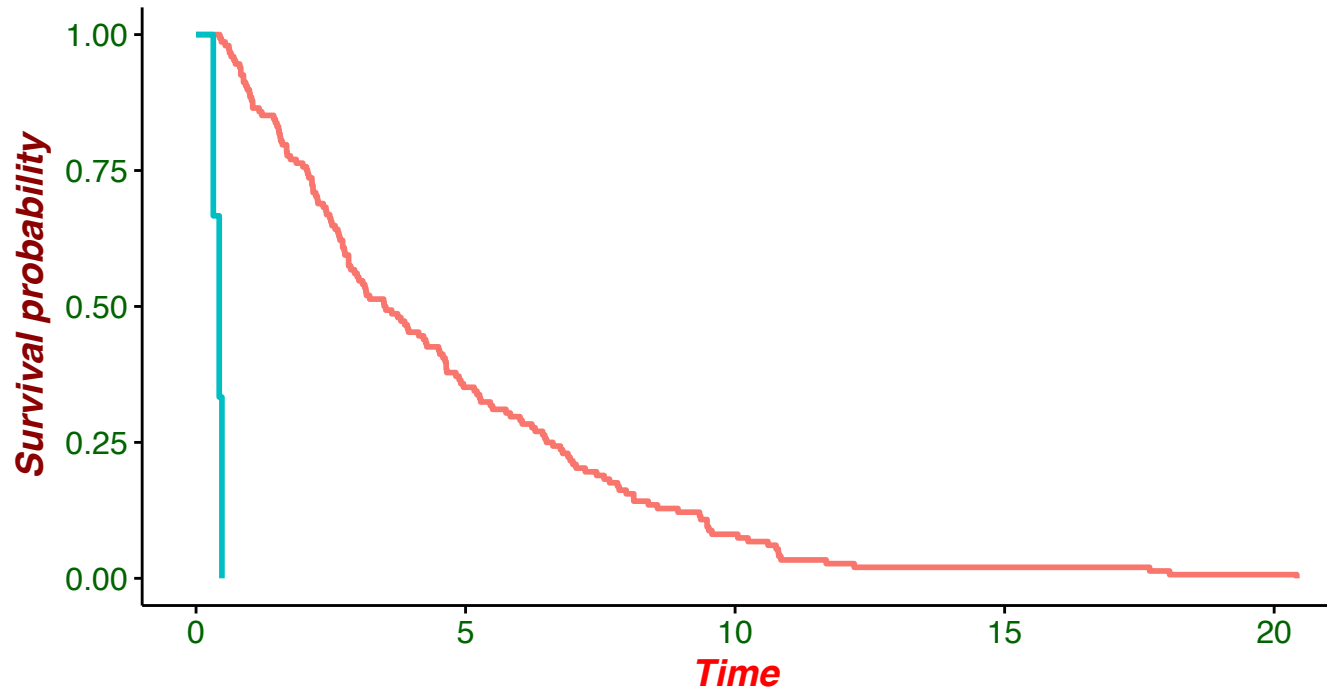

Supplement: Supplementary file 3 — The Kaplan–Meier estimator using top 35 mutated genes. The Kaplan–Meier curves using 35 mutated genes selected by the univariate regression as predictors displayed two very unbalanced survival risk groups. (PDF 15 kb) [file 12920_2018_419_MOESM3_ESM.pdf]

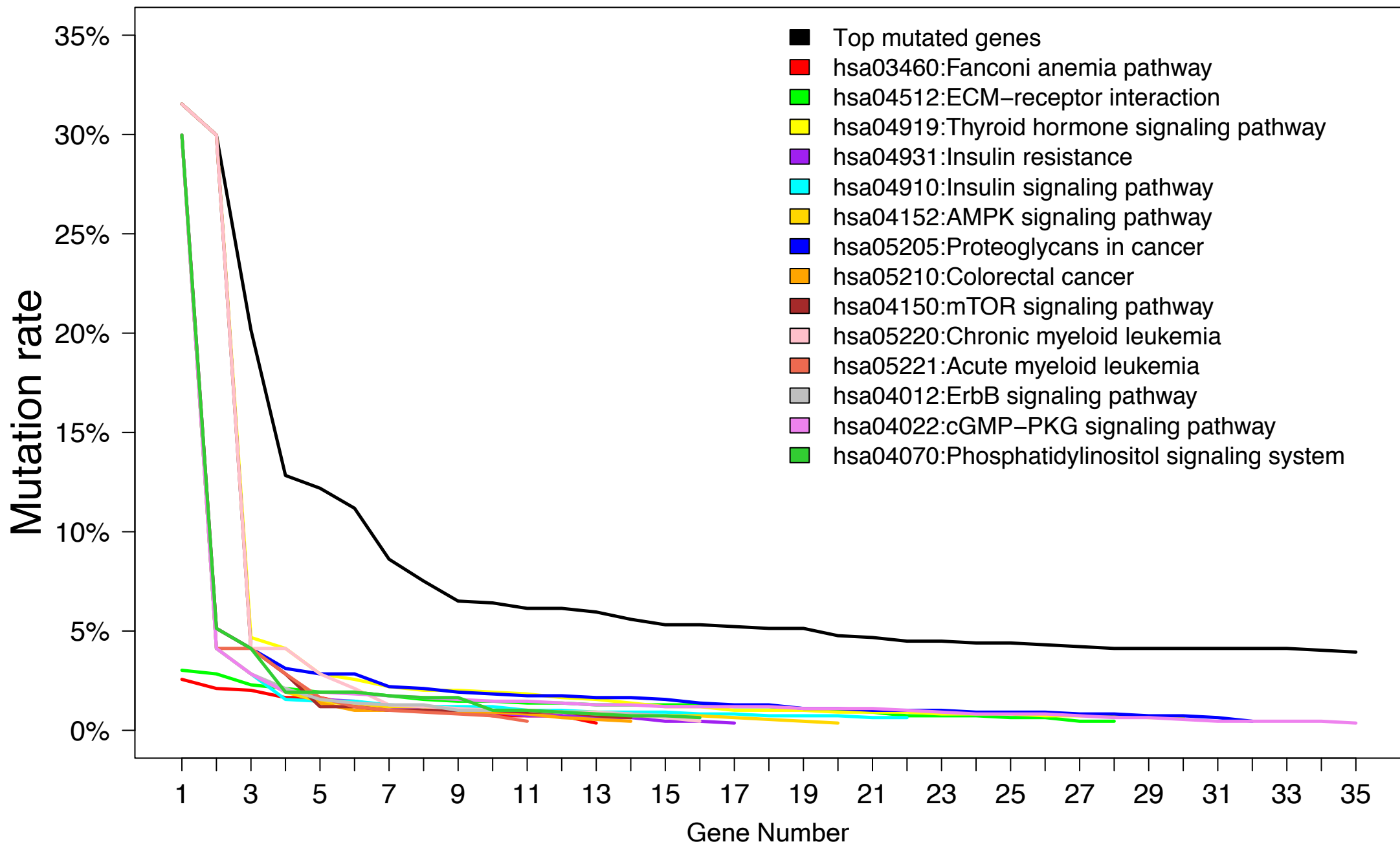

Supplement: Supplementary file 4 — The comparison of mutation rates for top mutated genes and 14 pathway genes. The mutation rates for 15 different gene sets, including one top-35-mutated gene set and 14 pathway-based gene sets. The genes in each set were ordered by their mutation rates. (PDF 36 kb) [file 12920_2018_419_MOESM4_ESM.pdf]

A

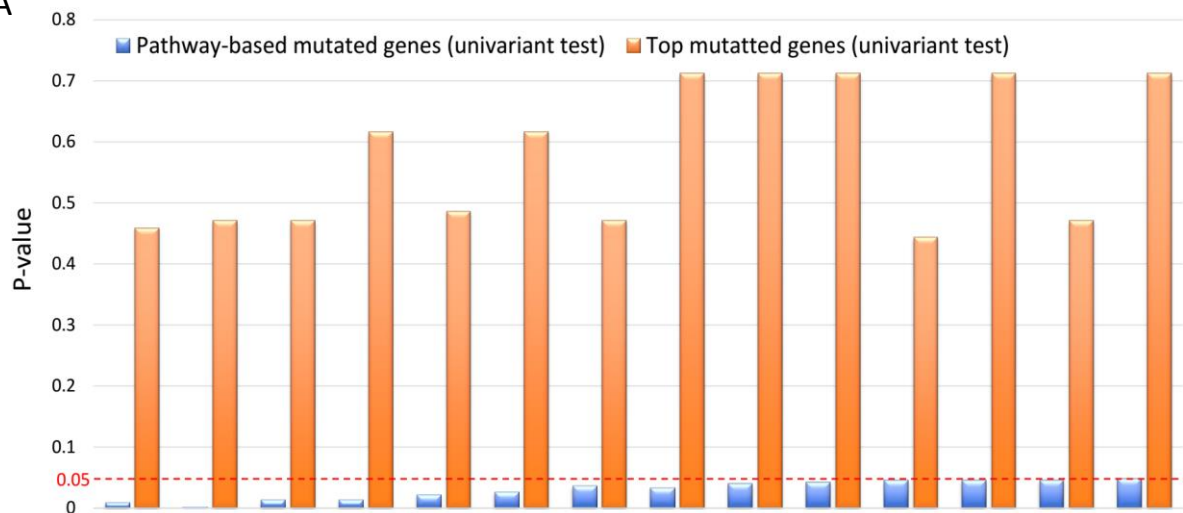

B

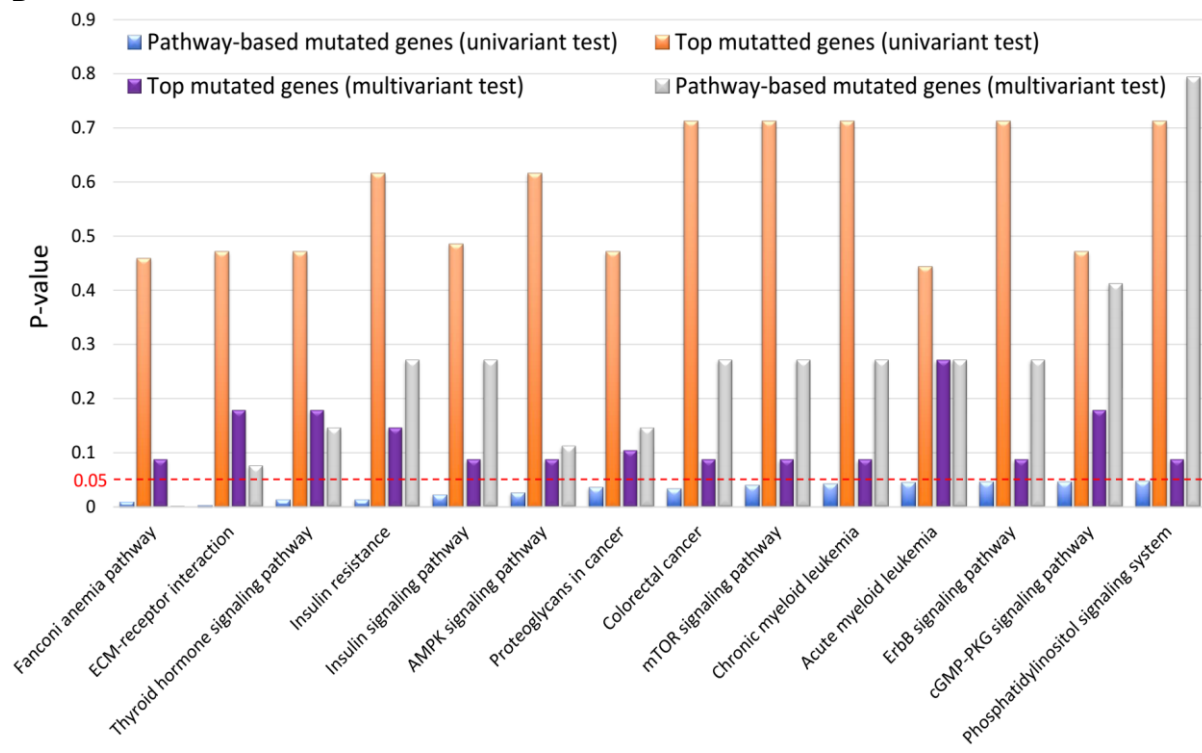

Supplement: Supplementary file 5 — The P-value of the Cox univariate regression analysis using different gene sets. (A) The P-values of the Cox univariate regression analysis for pathway-based genes (blue) and top mutated genes (orange). (B) The P-values of univariant test and multivariant test for pathway-based genes (blue and grey) and top mutated genes (orange and purple). (PDF 344 kb) [file 12920_2018_419_MOESM5_ESM.pdf]
